# Climate controls on snow reliability in French Alps ski resorts

**DOI:** 10.1038/s41598-019-44068-8

**Published:** 2019-05-29

**Authors:** P. Spandre, H. François, D. Verfaillie, M. Lafaysse, M. Déqué, N. Eckert, E. George, S. Morin

**Affiliations:** 1 Univ. Grenoble Alpes, Irstea, UR LESSEM, 38000 Grenoble, France; 2 Univ. Grenoble Alpes, Université de Toulouse, Météo-France, CNRS, CNRM, Centre d’Études de la Neige, 38000 Grenoble, France; 3 Barcelona Supercomputing Center, Barcelona, Spain; 4 CNRM, Université de Toulouse, Météo-France, CNRS, 31000 Toulouse, France; 5 Univ. Grenoble Alpes, Irstea, UR ETNA, 38000 Grenoble, France

**Keywords:** Climate change, Cryospheric science

## Abstract

Ski tourism is a major sector of mountain regions economy, which is under the threat of long-term climate change. Snow management, and in particular grooming and artificial snowmaking, has become a routine component of ski resort operations, holding potential for counteracting the detrimental effect of natural snow decline. However, conventional snowmaking can only operate under specific meteorological conditions. Whether snowmaking is a relevant adaptation measure under future climate change is a widely debated issue in mountainous regions, with major implications on the supply side of this tourism industry. This often lacks comprehensive scientific studies for informing public and private decisions in this sector. Here we show how climate change influences the operating conditions of one of the main ski tourism markets worldwide, the French Alps. Our study addresses snow reliability in 129 ski resorts in the French Alps in the 21st century, using a dedicated snowpack model explicitly accounting for grooming and snowmaking driven by a large ensemble of adjusted and downscaled regional climate projections, and using a geospatial model of ski resorts organization. A 45% snowmaking fractional coverage, representative of the infrastructures in the early 2020s, is projected to improve snow reliability over grooming-only snow conditions, both during the reference period 1986–2005 and below 2 °C global warming since pre-industrial. Beyond 3 °C of global warming, with 45% snowmaking coverage, snow conditions would become frequently unreliable and induce higher water requirements.

## Introduction

Grooming and snowmaking are standard snow management components in ski resorts, used to reduce the impact of the interannual variability of natural snow conditions, but are also increasingly regarded as methods to adapt to observed and projected decline of natural snow conditions in mountainous areas^1–4^. Climate projections of natural snow conditions, which are regularly viewed as alarming for the future of ski resorts^3,5,6^, are thus insufficient to address the impact of climate change on ski resort operations^2,7^. While, on the demand side, behavioural research studies have demonstrated the importance of natural snow conditions on skier participation and visitation in North America and Europe^5,8,9^, assessing snow reliability on the supply side needs to account for snow management^2,4,7^. Because snowmaking intrinsically depends on meteorological conditions (wet-bulb temperature and wind speed) and on the availability of water resources used to produce snow, it is itself climate sensitive. There is a growing number of studies addressing climate change impacts on ski resorts operations accounting for snowmaking^2^, although several limits need to be considered. On the technical side, the models used to simulate snow, with or without snowmaking, generally represent snow processes in a very simple way and rely on empirical temperature-based relationship to compute the melt rate^10^. They also ignore the key role of grooming in densifying and mixing snow layers (including natural and machine-made snow layers), with implications for the physical behaviour of snow on ski slopes^11^. Natural snow conditions and machine made snow production time are often handled independently, although snow conditions on ski slopes are a joint product of these two components^12^. Last, in-depth studies have been performed, but they operate at the local scale^13^ without easy upscaling at the regional level. Conversely, large-scale studies often address snow reliability in ski resorts using a very limited number of locations (often only one, either at the base or in the middle of the elevation range) characterizing their geographic position and elevation, with potentially strong biases due to the imperfect representation of ski resorts spatial organization and elevation, aspect and slope of the ski slopes^10,12^. It is thus needed to develop and implement methods, which represent snow management in a detailed, consistent and integrative way, but can still operate at the regional scale and be applied to a large number of ski resorts.

The climate sensitivity of French winter tourism has only recently^14^ received specific attention although France is a major ski destination. Indeed, the European Alps represent 43% of worldwide skier visits mainly shared between France and Austria which are both in the top 3 ski destinations in the world with more than 50 millions of skier visits each year (51.1 millions for the snow season 2016–2017, more than 15% of the worldwide skier visits)^15,16^. The French Alps are the main ski area in France with 84% of national ski-lift facilities^17^. Tourism services support a large part of direct or indirect employment in mountain communities^18^. Far-reaching political decisions have been made over the past decades in terms of the support offered by public authorities to this economic sector. As an example, subsidies for ski resorts considered at too low elevation to be sustainable in the long term were cut in the past^17^, and massive support of snowmaking equipment regardless of an assessment of the long-term climatic feasibility was implemented after a change of regional political orientation. This policy has recently been criticized by the French Court of Auditors^19^, on the basis of natural snow depth records and climate projections. This illustrates the conundrum faced by public and private decision makers at national, regional and local levels in part due to the lack of appropriate information.

In order to quantify the snow conditions in French Alps ski resorts under present and future climate conditions we used state-of-the-art climate reanalysis and projections adapted to the mountain environment^20–22^ to drive a dedicated snowpack model explicitly accounting for grooming and/or snowmaking on ski slopes^11,23^. Snow conditions were further combined with spatial representations of ski areas^24^ (see Methods).

**Figure 1:**
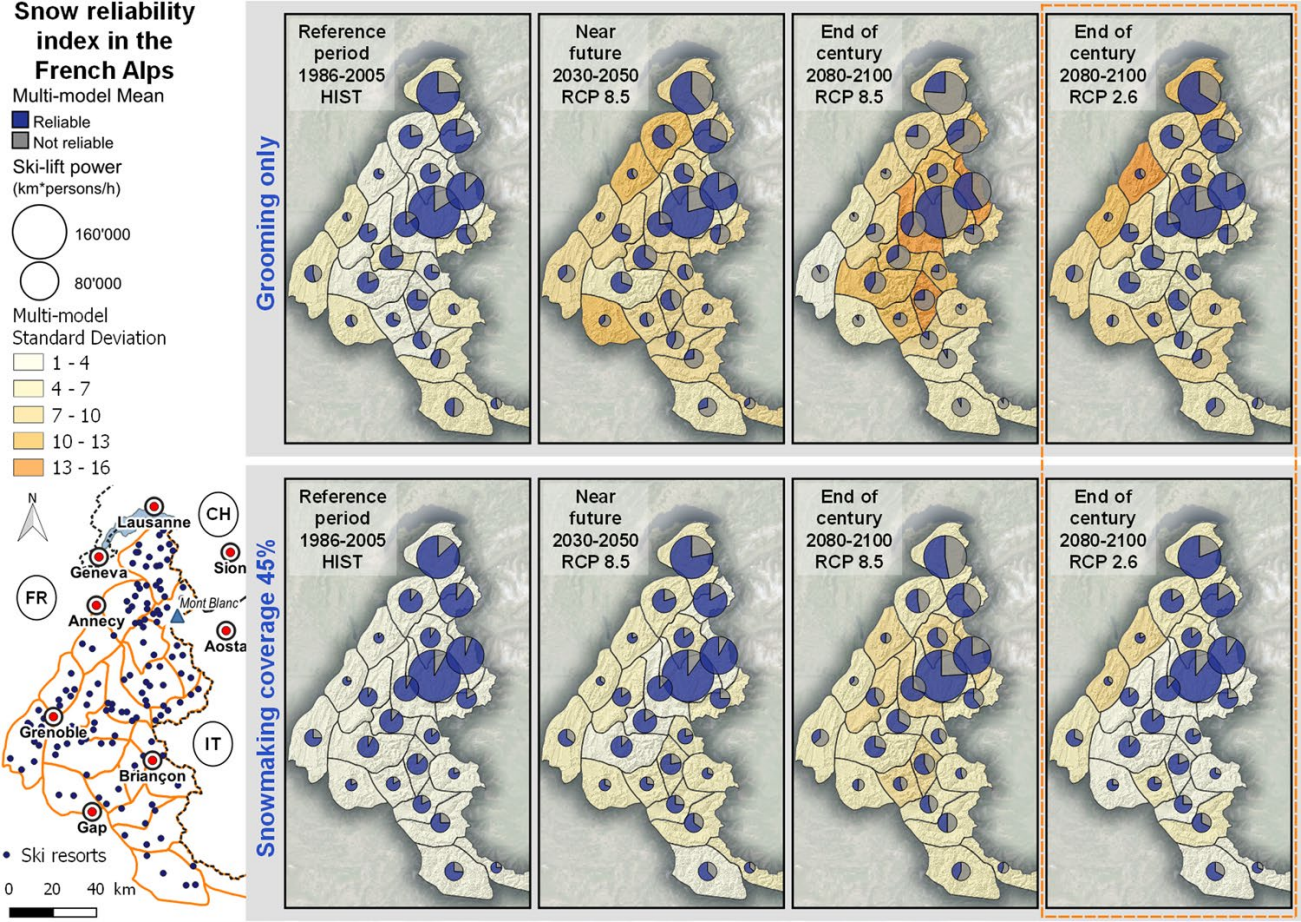
Mean and standard deviation across multiple model estimates of 20-yr averages of the fractional snow reliability index for groomed snow conditions (top) and groomed snow conditions plus a 45% snowmaking coverage (bottom), for the reference period (1986–2005), the near future (2030–2050, RCP 8.5) and the end of century (2080–2100, RCP 8.5 and RCP 2.6, surrounded by red dashed lines) for the 23 massifs of the French Alps. Pie diagrams display the 20 year multi-model massif-scale snow reliability index, the diameter being proportional to the total ski-lift power within each massif. Background colors display the standard deviation around the mean. Situation of the 129 ski resorts covered in the present study and of the main cities in the French Alps (lower left).

The chain of models accounts explicitly for operational snow management methods^25^, including past and projected evolution (from 0% in 1985 to 45% in 2025^26^) of the fraction of ski slope surface area equipped with snowmaking facilities (referred to as the snowmaking coverage, %). We defined and computed a snow reliability index for every single ski resort for Christmas and February school break holidays i.e. two key periods for winter sports (see Methods). When aggregated at the scale of the entire French Alps (129 ski resorts sample, Fig. 1), our method covers 96% of the French Alps ski-lift infrastructures. The simulations provide estimates of water volumes required for snowmaking under given climate conditions and snowmaking coverage.

**Figure 2:**
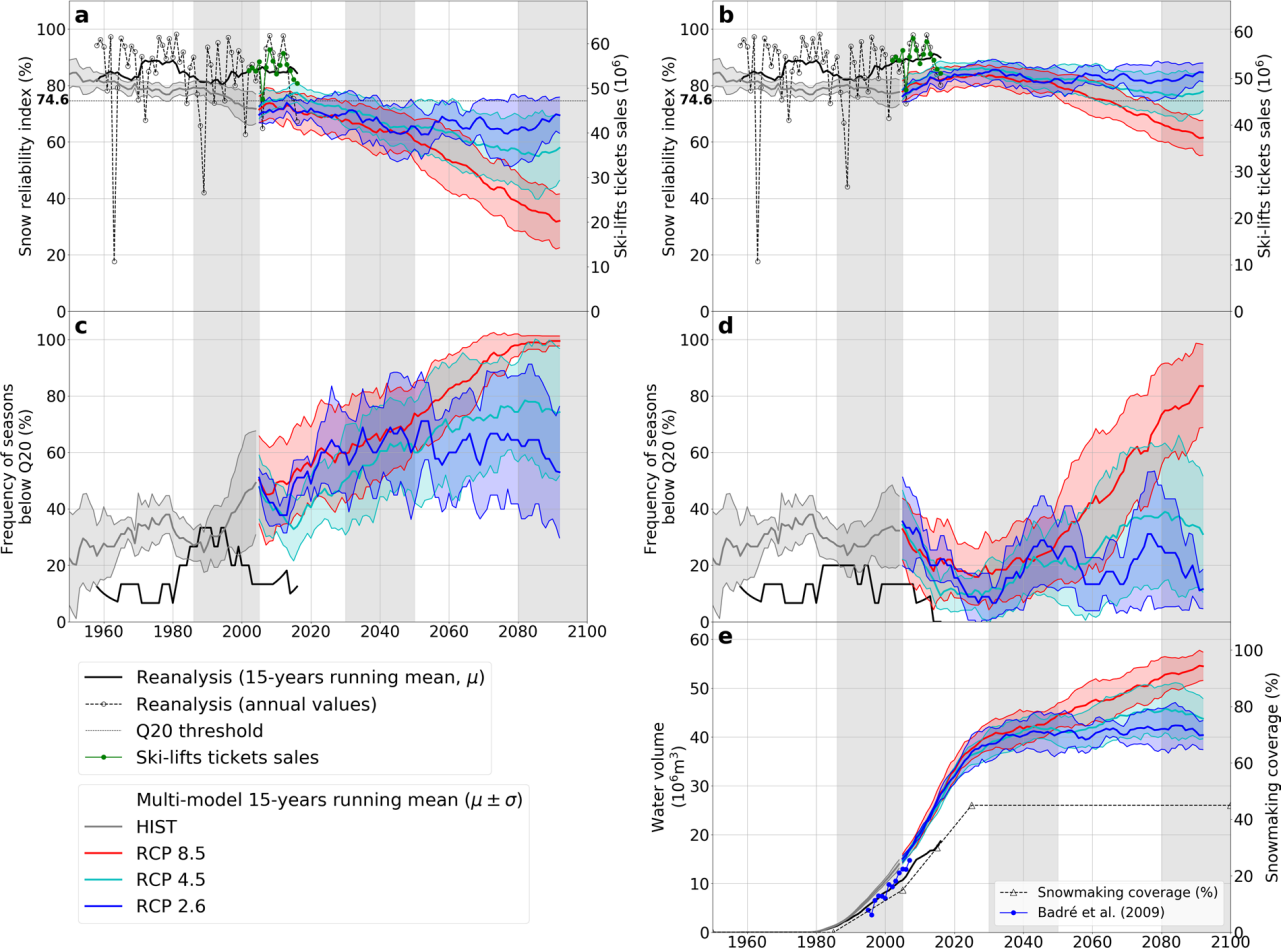
Time evolution of the snow reliability index (including reanalysis annual values) for (**a**) groomed snow conditions and (**b**) groomed snow conditions with snowmaking, including independent estimates of annual ski-lift ticket sales. Time evolution of the frequency of snow seasons exhibiting fractional snow reliability values lower than the snow scarcity threshold Q20 for (**c**) groomed snow conditions and (**d**) groomed snow conditions with snowmaking. (**e**) Water demand associated to the production of snow, including independent estimates of the water volumes used for snowmaking^27^. All figures display the 15-yr average of the reanalysis and multi-model mean and standard deviation of 15-yr averages for historical and future climate scenarios.

Snow-scarce seasons were defined over the historical period (1958–2016) based on a snow reliability index threshold of 74.6% (Q20), corresponding to the 20% lowest index values under groomed snow conditions only (no snowmaking). Over the reference period (1986–2005), four seasons show snow reliability index lower than the Q20 threshold: 1988–1989, 1989–1990, 1992–1993 and 2001–2002. The three first ones are often referred to as the “early 1990’s snow-scarce seasons”. They revealed for the first time the dependency of Alpine winter tourism to natural snow conditions and climate variability, and played a large role for the inception and development of snowmaking in the French Alps^26^. Snow-scarce winter seasons occurred before, in 1963–1964 and 1972–1973, without the same impact on this emerging economic sector at the time. Since 2005, two additional snow seasons showed snow reliability index lower than the Q20 threshold: 2006–2007 and 2016–2017. Using a time variable snowmaking coverage value^26^ (Methods) and reanalysis driving data from 2001 to 2016, the annual snow reliability index and ski-lift ticket sales are significantly correlated, with correlation coefficients of 0.91, 0.89 and 0.87 for natural snow, grooming-only and managed snow conditions, respectively (Fig. 2b). The slope of the relationship, i.e. the percentage increase in normalized skier days for a 1% increase of the reliability index amounts 0.57%, 0.68% and 1.02%, respectively. Although the correlation is slightly better between natural snow conditions and ski-lifts tickets sales than accounting for snow management, the quantitative relationship between the two, also referred to as the elasticity, is more consistent in the case where grooming and snowmaking are taken into account. Simulation data from 1995 to 2008 show a good agreement (within 20%) with the water volume requirements estimated by Badré *et al*.^27^.

**Figure 3:**
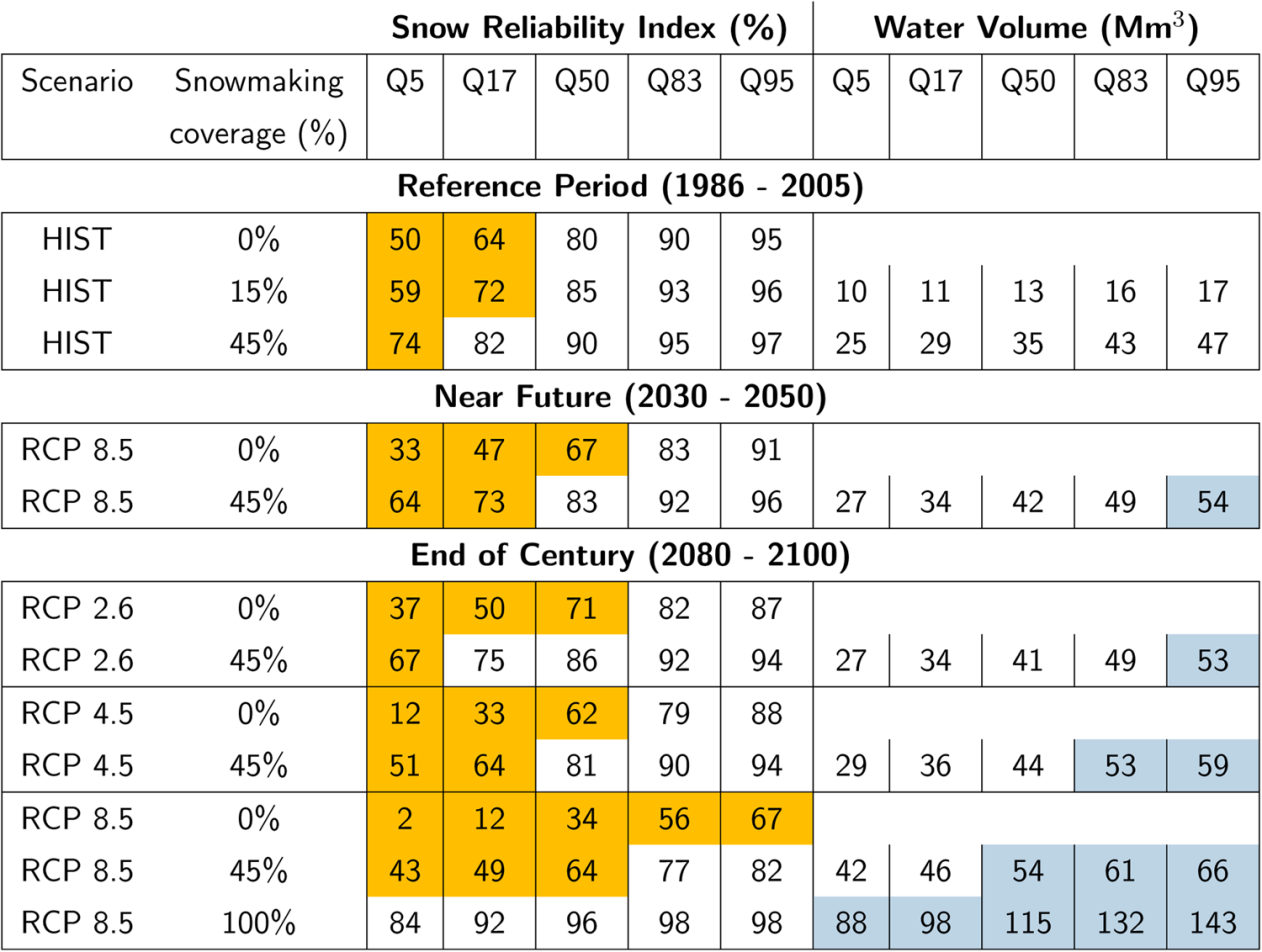
Evolution of the snow reliability index (%) and water volume (Mm^3^) for snowmaking of French Alps ski resorts for the reference period 1986–2005, near future (2030–2050) and end of century (2080–2100), depending on the RCP scenario and the snowmaking coverage (in % of total ski slopes surface area). Snow reliability values below the snow scarcity threshold (Q20) are highlighted in orange. Water volumes over three times the 95% percentile value (Q95) of the reference period are highlighted in blue. Note that years contributing to a given quantile for the snow reliability index do not necessarily contribute to the same quantile in terms of water volume for snowmaking.

At the scale of the entire French Alps, our analysis indicates that snowmaking indeed plays a major role for snow reliability in ski resorts. Based on climate model outputs from the historical experiments (HIST) during the reference period 1986–2005, the median annual reliability index would increase by 10%, from 80% under no snowmaking to 90% under 45% snowmaking coverage. Figures are even more compelling for the scarcest snow seasons (Q5), showing a 50% reliability index without snowmaking, rising with snowmaking to 59% (15% snowmaking coverage) up to 74% (45% snowmaking coverage). The latter value corresponds almost to the Q20 threshold, computed without snowmaking (Fig. 3). Snowmaking does not make a significant difference for years with abundant snow conditions (Fig. 3). Median water requirements for snowmaking with 15% snowmaking coverage amount 13 Mm^3^, ranging from 10 Mm^3^ (Q5) to 17 Mm^3^ (Q95, Fig. 3). Water requirements are primarily driven by increases in snowmaking coverage for the period from 1985 to 2025 (Figs 2e and 3). During the time period of overlap, the results obtained from reanalysis data and for multi-model mean and deviation of historical climate model experiments (HIST) exhibit some differences. The latter shows lower snow reliability, higher frequency of snow-scarce seasons, and higher water requirements, although individual meteorological variables were bias-adjusted over the historical period. Discrepancies might therefore be related to potential biases of the multivariate distribution of the meteorological variables produced by the adjustment and downscaling method^21^. This might result in potential nonlinear effects due to multiple dependencies especially on temperature, relative humidity, precipitation and wind-speed. A second reason might be that the reanalysis data describe one of the many possible realizations of the climate system, several of which are also probed by the ensemble of GCM/RCM pairs (only forced by greenhouse gases emissions), in a manner which does not guarantee perfect overlap between the two due to climate internal variability^22^. However, comparing reanalysis and climate modeling experiments provides context for the latter and does not affect the analysis of the future climate trends.

Without snowmaking, the frequency of challenging snow seasons is projected to rise significantly in the near future (2030–2050), regardless of the Representative Concentration Pathway (RCP, Fig. 2c). The median reliability index would stand below the Q20 threshold computed over the historical period (Fig. 3). Challenging seasons encountered once every five years in the reference period would then occur once every two years, which has strong implications for ski resort operations. At the projected snowmaking coverage of 45%, however, the 2030–2050 median snow reliability under RCP 8.5 would equate the figure for groomed-only snow during the reference period 1986–2005. Compared to the situation with 45% snowmaking coverage during the reference period, the snow reliability is projected to decrease for all percentiles of the annual reliability values (Fig. 3). This may be related to the decrease of natural snow conditions at all elevations combined to the higher occurrence of snow-scarce seasons with low production at lower elevations related to the lack of suitable meteorological conditions for snowmaking. Because of both increased requirements for snowmaking due to reduced natural snow conditions, and increasing snowmaking coverage, water requirements are projected to increase, with median requirements under a 45% snowmaking coverage rising from 35 to 42 Mm^3^ from 1986–2005 to 2030–2050 (RCP8.5), and from 47 to 54 Mm^3^ (Q95). Increased natural snow scarcity accounts for an additional 10 to 20% snowmaking requirements if considering a constant snowmaking coverage between the reference period and the near future. Most of the changes in water requirements for snowmaking may thus remain highly dependent on the evolution of the snowmaking coverage (Fig. 2).

For the end of the century (2080–2100), the evolution of snow conditions is projected to be highly dependent on the RCP (Figs 1, 2 and 3). Compared to 2030–2050, the groomed snow reliability index is projected to remain steady (RCP 2.6), slightly decrease and stabilize (RCP 4.5) or continuously decrease (RCP 8.5). The reliability index of the most abundant snow seasons (Q95) is projected to reach values of 88% and 67% for RCP 4.5 and RCP 8.5, respectively, which spans the range between the Q17 and Q83 without snowmaking for the reference period 1986–2005. Assuming a 45% snowmaking coverage, the median snow reliability index under RCP 4.5 would correspond to the median reliability index of groomed-only snow during the reference period. Under RCP 8.5, the median snow reliability index with a 45% snowmaking coverage would correspond to the Q17% of the reliability index of groomed-only snow during the reference period.

The median, and higher percentile (Q95), of water consumption for snowmaking would amount 44 and 59 Mm^3^ for RCP 4.5, respectively, and 54 and 66 Mm^3^ for RCP 8.5, respectively. At the scale of the entire study area, the increasing occurrence of scarce snow seasons with low production due to the lack of suitable meteorological conditions is compensated by the increasing requirements at higher elevations. Hypothetically, if snowmaking coverage keeps increasing until 100% by the end of the century, the snow reliability index would range between 84% (Q5) and 98% (Q95), i.e., higher than median groomed-only snow reliability for the reference period, corresponding to annual water consumptions ranging between 88 Mm^3^ (Q5) and 143 Mm^3^ (Q95) with a median of 115 Mm^3^. This would correspond to a factor 9 increase of water consumption compared to median requirements for a 15% snowmaking coverage over the reference period 1986–2005. Similar to the current and future situation with 45% snowmaking coverage, detailed studies at the local scale are required to assess whether water could be available to fulfill this specific demand.

Our analysis focuses on the snow reliability on ski slopes and corresponding water requirements for snowmaking at the scale of the entire French Alps, and provides critical information on the future operating capability of the French Alps ski tourism sector as an integrated production facility. However, impacts of climate change on ski resort operating conditions are heterogeneous in space (location, elevation). By design, our approach encompasses most geospatial characteristics of ski resorts, which need to be considered to aggregate information from the entire skiable area into the ski resort itself (elevation, aspect, slope, location of snowmaking facilities, location of ski lifts along with their transportation capacities etc.; see Methods). Because the location and geospatial characteristics of ski resorts in the French Alps is heterogeneous, this overall strength, in terms of realism at the resort level, makes it more difficult to draw general conclusions than methods focusing on snow reliability elevations regardless of the locations and spatial characteristics of ski resorts^28^. Nevertheless, several key findings can be summarized at the sub-regional level. Firstly, under current climate conditions, Fig. 1 indicates that the snow reliability of French Alps ski resorts is highly heterogeneous, with higher reliability, with or without snowmaking, in the North-East of the French Alps, mostly due to the fact that the large ski resorts in such areas are at typical higher elevation, than in the West and South. Under RCP8.5 at the end of the century, the heterogeneity remains, with snow reliability index values still above 50% on average without snowmaking in the same high-elevation large ski resort area in the North East, while the snow reliability index is largely lower than 50% for most other areas. A similar contrast is visible with 45% snowmaking coverage. In terms of the trend within each sub-region and elevation range, the pattern is generally similar, due to the fact that the trend analysis is performed on an indicator (frequency of seasons below the Q20 threshold) which is normalized to the snow reliability under the current climate. Even ski resorts with a mean elevation above 2,000 m above sea level undergo a similar increasing trend of water requirements and decreasing trend of snow reliability, compared to the reference situation under current climate. However, the choice of using a Q20 reliability index does not preclude whether situations above this threshold value are fully appropriate for skiing. It may be the case, especially in low elevation ski resorts, that the Q20 value is already lower that the threshold for minimum operating conditions. Detailed assessments analyzing results for individual ski resorts are required for such deeper exploitation of the data set used in this study.

**Figure 4:**
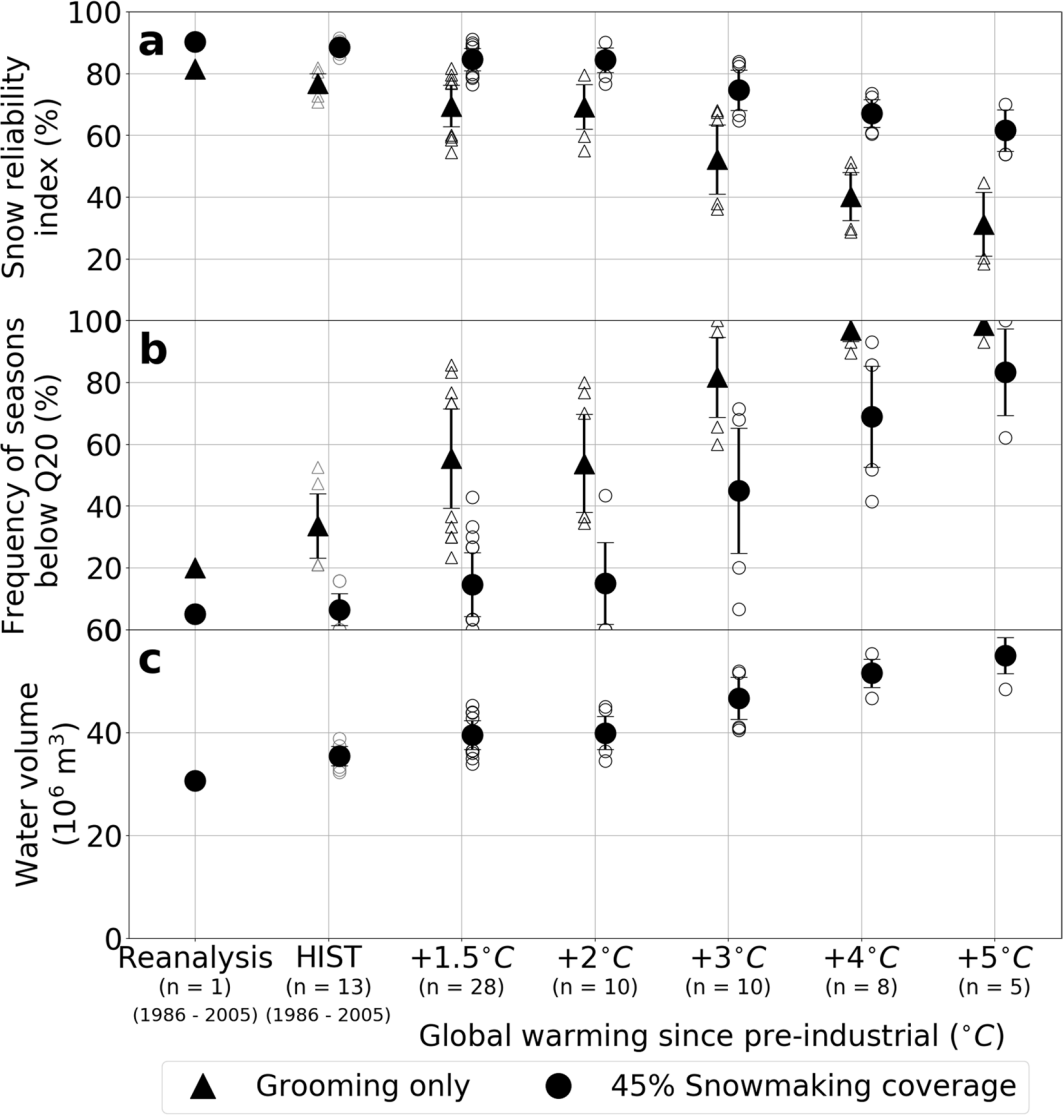
Relationship to global warming level of (**a**) the snow reliability index, (**b**) the frequency of snow seasons below the snow scarcity threshold (Q20) and (**c**) water demand associated to the production of snow, accounting for grooming only (0% snowmaking coverage) and a 45% snowmaking coverage. The global surface air temperature change was computed with respect to the pre-industrial period (1851–1880). The mean value, standard deviation (error bars) and outliers are shown (see Methods). n is the number of GCM/RCM pairs within a given temperature bin.

At the scale of the entire French Alps, our results can be analyzed as a function of the level of global warming since the pre-industrial period, in complement to future scenarios. Using a multi-scenario time sampling approach^29^, Fig. 4 provides critical information that is policy-relevant in a context of climate change adaptation and mitigation, including for an analysis of the impact of a warming of 1.5 °C and 2 °C. Compared to the reference period 1986–2005, which corresponds to about 0.62 °C global warming, 1.5 °C and 2 °C global warming translate approximately into the same consequences in terms of groomed-only or 45% snowmaking snow reliability in the French Alps. At 1.5 °C and 2 °C global warming, a 45% snowmaking coverage provides generally higher snow reliability than the groomed-only reference period situation. At 3 °C global warming and beyond, groomed-only reliability is projected to drop markedly with a mean frequency of snow-scarce seasons (i.e. below Q20 threshold) reaching 80% of years and beyond. This global warming level marks a turning point for snowmaking too: in this case, the frequency of occurrence of snow-scarce seasons becomes higher than the groomed-only reference period, and this frequency increases markedly for further degrees of warming. Our analysis of the regional warming level shows an enhancement factor, for the time period from December to April, on the order of 1.10 °C of regional warming per °C of global warming (computed over similar 30-years time periods). At 1500 m elevation, global warming levels of 1.5 °C, 2 °C and 4 °C since pre-industrial time correspond to local warming with respect to the reference period 1986–2005 of 0.9 ± 0.1 °C, 1.2 ± 0.1 °C and 3.9 ± 0.4 °C, respectively. Such findings are consistent with literature estimates^30^.

 This study introduces objective information on the snow reliability of ski resorts in the French Alps, based on a numerical and statistical modeling framework featuring an unprecedented level of detail, which was applied systematically over a region harboring more than 100 ski resorts. We considered current and conventional snow management techniques (see Methods), and neither potential disruptive transformations of ski tourism offer, nor major changes of snow management techniques were accounted for. Local deviations to main snow management strategies might also be possible and could only be addressed in specific studies. Notwithstanding regional differences, the response is expected to be similar for many ski resorts located in the European Alps and North America^2,3^. Last, while our analysis accounts explicitly for snowmaking and quantifies water requirements, it does not provide information on whether such water amounts are available, at the time of the season when there are required. Additional studies would be needed to address this critical issue and address comprehensively the water cycle in and around ski resorts including potential conflicts in the use of the available resource under current and future climate conditions. They could be developed based on the methodology implemented in this study.

Although this study introduces an analysis of climate controls on snow reliability at the scale of French Alps ski resorts as a whole, the underlying data could be used for detailed studies, thereby providing the most objective and rigorous framework possible for informing public debates on critical matters in the mountain environments, regarding tourism development, water and energy use, and more generally human well-being and habitability of mountain areas, either at the large scale reported in this study or a the scale of one or several individual ski resorts to take into account local specificities. There remains a need for a comprehensive, continental if not global-scale, assessment of the impact of climate change on ski resort operations, using an homogenized framework^2^. Such information is critical to provide policy-relevant information supporting public and private decisions, spanning all appropriate time scales of the decision for planning large-scale infrastructure such as ski lifts or snowmaking coverage. Additional constraints on winter tourism (lowering demand due to a shift in recreational activities or increased travel and/or energy price) might also be more threatening to ski resort operations than the mid to long-term climate trends^5,8,9^, and should be investigated in combination to climate impacts on the supply side.

## Methods

### Modeling of snow ski slopes

The Crocus Resort version of the multilayer snowpack model SURFEX/ISBA - Crocus^23^ allows taking into account the grooming and snowmaking effects on snow properties so as to provide simulations of snow conditions on ski slopes^11^. The model explicitly simulates physical processes within the snowpack (e.g. snow metamorphism, compaction, heat conduction, melt/freeze and liquid water percolation) and at its boundaries with the overlying atmosphere (full surface energy and mass balance) and underlying ground. The natural snow version of Crocus (without snow management) performed well in a recent snowpack model intercomparison exercise^31^. The physical impacts of grooming are simulated (enhanced densification and mixing of layers) and machine-made snow can be added to the snowpack specifying the production rate and conditions for triggering the production (wet-bulb temperature threshold, target quantity or target snow depth), with management rules depending on the periods of the winter season, accordingly to current snowmaking practices in ski resorts^25,32–34^. Between November 1 and December 15, a 30 cm deep “base layer” (150 kg m^−2^, corresponding to 30 cm of snow at 500 kg m^−3^ typical density on ski slopes) is produced, weather conditions permitting, regardless of natural snowfalls during the period. Between December 15 and February 28, snow is produced if meteorologically possible so as to maintain a total snow depth of 60 cm. After March 1, no more snow is produced. The snowmaking instantaneous production rate was set to 1.2 10^−3^ kg m^−2^ s^−1^, i.e. 4.32 kg m^−2^ h^−1^ ^25^. The wet-bulb temperature threshold to trigger snowmaking was set to −2 °C i.e. the maximum temperature ensuring the technical feasibility of conventional snowmaking^35^. The wind speed threshold value was set to 4.2 m s^−1^ ^11^. A constant water loss during the snowmaking process of 40% of the total water volume^36,37^ was taken into account i.e. it is assumed that 60% of the water used for snowmaking was converted to snow.

### Climate projections

The SAFRAN meteorological reanalysis dataset at hourly time resolution was used as a reference over the historical period 1958–2016^38^. This reanalysis is based on geographic areas assumed to be homogeneous (massifs) within which atmospheric variables depend only on elevation (provided by 300 m steps), slope angle and aspect. SAFRAN reanalysis data from 1980 to 2010 were used by the ADAMONT method^21^ as an observation database to statistically adjust to the massif-scale by elevation bands, and disaggregate to hourly time resolution, the surface atmospheric fields of the ensemble of 13 pairs of global and regional climate models from the EURO-CORDEX database^20,39^ used in Verfaillie *et al*.^22^. This spans historical (1950–2005) and future (2005–2100) time periods for scenario RCP 4.5, RCP 8.5, and for a subset of 4 GCM/RCM pairs for RCP 2.6^22^.

### Spatial representations of ski resorts

The geographical location of ski lifts was used to infer an estimate of ski slope in ski resorts^17,24^. The French *“Services Techniques de Remontées Mécaniques et Transports Guidés”* (STRMTG) manages an exhaustive database of cable transportation equipments in France, which includes technical characteristics of each ski lift. This includes the ski lift power (SLP) of each ski lift, which is defined as the product of the elevation difference between the bottom and the top of a ski lift (in km) and its flow of persons per hour (pers h^−1^). It is expressed in pers km h^−1^. These data are completed with geographical information from the database BDTOPO (25 m of resolution) developed by the French Geographical Institute (IGN). We used the representation of ski slopes named gravitational envelopes^17^, derived from ski-lift locations and defined as the ensemble of points which can be reached from both the bottom and the top of a ski lift. We considered three different snowmaking coverage of ski areas: 15%, 30% and 45%, corresponding to the actual value in 2003^28^, 2015 and the expected value in 2025^26^, respectively. Their spatial distribution of snowmaking units is inferred from the altitudinal and geospatial structure of ski resorts, allowing to compute a snowmaking envelop for each value of the snowmaking coverage^17^. The surface area of the ski slopes was estimated from the total surface area of the gravitational envelope using a ratio of 11% obtained from a previous study^17^. Additional details can be found in Spandre, 2016^40^.

### Computation of snow indicators

The simulated snow conditions for each day are associated to every pixel within the gravitational envelope of a given ski resort based on its main geographical features: elevation, slope angle, slope aspect and whether the pixel is within the snowmaking envelope. A daily snow reliability index (%) is then defined as the fraction of the surface area of the gravitational envelope with a minimum quantity of snow for skiing (100 kg m^−2^, corresponding to 20 cm of snow at 500 kg m^−3^ typical density on ski slopes). The snow reliability index of a ski resort is derived from the means of daily indices of Christmas (20 December to 5 January) and February (5 February to 5 March) holidays, with a 17% weight on the Christmas period and 83% weight on the February period. The use of these time periods and this partitioning between Christmas and February was found to maximize the statistical relationship between the obtained snow reliability index and ski lift tickets sales at the scale of the French Alps^40^. The computation of the snow reliability index for all ski resorts of the French Alps is obtained as an average weighted by the SLP of individual ski resorts^17,24^. Based on the snow reliability index computed with the SAFRAN reanalysis over the historical period (1958–2016) for groomed snow conditions (no snowmaking), a snow scarcity threshold was defined as the percentile 20% of annual values (Q20). A bootstrap method was applied (n = 10 000 re-sampling of snow reliability index values) to provide a 90% confidence interval for the Q20. The lower boundary of the 90% confidence interval was defined as the snow scarcity threshold Q20 so as to be sure to isolate snow seasons with marked snow scarcity. The frequency of seasons with snow reliability index lower than the Q20 were computed based on climate model experiments and further referred to as the frequency of snow-scarce seasons. The annual water volume for snowmaking was computed for every ski resort based on the simulated amount of machine-made snow for every pixel within the snowmaking envelope, depending on the snowmaking coverage (15%, 30% and 45%). The annual French Alps water volume for snowmaking is the sum of all ski resorts water volume, expressed in millions of cubic meters (Mm^3^). For every winter season beyond 1985, we assumed a linear evolution of the snowmaking coverage between 1985 (0%) and 2005 (15%), between 2005 (15%) and 2015 (30%), and between 2015 (30%) and 2025 (45%). Beyond 2025, we considered that the snowmaking coverage would no longer increase (45%). For any given winter season, the snow reliability index, the frequency of snow-scarce seasons and the water volume were computed based on the simulated data for 15, 30 and 45% snowmaking coverage and on the value for this specific winter season (linear interpolation).

### Variability, uncertainties and relationship to global warming

The time evolution of the snow-related indicators (fractional snow reliability index, frequency of seasons below the Q20, water volume for snowmaking) was computed for groomed snow conditions with and without snowmaking, for historical and future climate scenarios. The 15-yr multi-model percentiles of annual values (5%, 17%, 50%, 83% and 95%, respectively referred to as the Q5, Q17, Q50, Q83 and Q95) were computed and are displayed in Fig. 3. This illustrates not only the long-term trend but also the interannual variability. The mean and standard deviation of multi model 15-yr averages were also computed (displayed on Fig. 2), thereby suppressing most of the effects of the interannual variability. This focuses on long-term trends and uncertainty on the climate modeling^22^ also illustrating the decadal variability that appears in our analysis. A similar approach was taken for Fig. 1 based on 20-year periods (1986–2005, 2030–2050 and 2080–2100). Relationships between snow-related indicators and global warming level were computed as 20-year averages for the reference period (1986–2005) and for three 30-year periods for the beginning of century (2011–2040), middle of century (2041–2070), and end of century (2071–2100), for all available GCM/RCM pairs and RCPs. For each GCM–RCM pair and a given RCP and period, the global surface air temperature change was computed with respect to the pre-industrial period (1851–1880) from the corresponding GCM run for the given RCP. The computed values of snow-related indicators were binned according to the corresponding global temperature changes (±0.25 °C): +1.5 °C, +2 °C, +3 °C, +4 °C, +5 °C.

## Data Availability

The datasets generated during and/or analysed during the current study are available from the corresponding author on reasonable request.

## Code Availability

The code of the ADAMONT v1.0 method is available as an open git repository after free registration at https://opensource.cnrm-game-meteo.fr/projects/adamont. The version of the open source code of SURFEX/ISBA-Crocus used in this study is available as a specific branch of an open git repository, after free registration, at https://opensource.cnrm-game-meteo.fr/projects/surfex_git2.
